# Chromosomal Evolution and Apomixis in the Cruciferous Tribe Boechereae

**DOI:** 10.3389/fpls.2020.00514

**Published:** 2020-05-28

**Authors:** Terezie Mandáková, Petra Hloušková, Michael D. Windham, Thomas Mitchell-Olds, Kaylynn Ashby, Bo Price, John Carman, Martin A. Lysak

**Affiliations:** ^1^CEITEC, Masaryk University, Brno, Czechia; ^2^Department of Biology, Duke University, Durham, NC, United States; ^3^Plants, Soils, and Climate Department, Utah State University, Logan, UT, United States

**Keywords:** apomixis, apospory, autopolyploidy, Cruciferae, descending dysploidy, karyotype evolution, North America, speciation

## Abstract

The mustard family (Brassicaceae) comprises several dozen monophyletic clades usually ranked as tribes. The tribe Boechereae plays a prominent role in plant research due to the incidence of apomixis and its close relationship to *Arabidopsis*. This tribe, largely confined to western North America, harbors nine genera and c. 130 species, with >90% of species belonging to the genus *Boechera*. Hundreds of apomictic diploid and triploid *Boechera* hybrids have spurred interest in this genus, but the remaining Boechereae genomes remain virtually unstudied. Here we report on comparative genome structure of six genera (*Borodinia*, *Cusickiella*, *Phoenicaulis*, *Polyctenium*, *Nevada*, and *Sandbergia*) and three *Boechera* species as revealed by comparative chromosome painting (CCP). All analyzed taxa shared the same seven-chromosome genome structure. Comparisons with the sister Halimolobeae tribe (*n* = 8) showed that the ancestral Boechereae genome (*n* = 7) was derived from an older *n* = 8 genome by descending dysploidy followed by the divergence of extant Boechereae taxa. As tribal divergence post-dated the origin of four tribe-specific chromosomes, it is proposed that these chromosomal rearrangements were a key evolutionary innovation underlaying the origin and diversification of the Boechereae in North America. Although most Boechereae genera exhibit genomic conservatism, intra-tribal cladogenesis has occasionally been accompanied by chromosomal rearrangements (particularly inversions). Recently, apomixis was reported in the Boechereae genera *Borodinia* and *Phoenicaulis*. Here, we report sexual reproduction in diploid *Nevada*, diploid *Sandbergia*, and tetraploid *Cusickiella* and aposporous apomixis in tetraploids of *Polyctenium* and *Sandbergia*. In sum, apomixis is now known to occur in five of the nine Boechereae genera.

## Introduction

Geographically well-defined clades provide ideal study systems for understanding the role of whole-genome duplications (WGDs, polyploidy) and chromosomal rearrangements in speciation and diversification. Frequently, a group of species confined to an island, mountain range, or (sub)continent is assumed to have originated in this region, perhaps following an earlier dispersal event from another part of the world (e.g., Linder and Barker, 2014; Givnish et al., 2016). With the advent of molecular phylogenetics, in many cases, the inferred monophyly of a group has confirmed its geographical determinant and helped to elucidate its origin as well as directionality of later migrations and dispersals (e.g., Cowie and Holland, 2008; Ogutcen and Vamosi, 2016; Huang et al., 2018; Carter et al., 2019; Kim et al., 2019). A geographically restricted clade might be supported by different synapomorphies, such as morphological traits, specific metabolic pathways, pollination syndromes, or a shared WGD, some falling in the category of rare genomic changes (RGCs, Rokas and Holland, 2000). Structural chromosomal changes may underlie incipient reproductive isolation inducing species splits and the evolution of separate gene pools, i.e., cladogenesis (Faria and Navarro, 2010). Dysploidal (i.e., chromosome number changes caused by fusions and fissions) as well as non-dysploidal (i.e., deletions, duplications, inversions, and translocations) chromosomal rearrangements can modify recombination frequency, gene expression, the duration of cellular processes (replication, mitosis, and meiosis), and the degree of infertility of heterozygous hybrids. Thus, some chromosomal rearrangements may precipitate lineage splitting yet occur within a monophyletic clade (Freyman and Höhna, 2017).

The economically important mustard family (3977 species in 351 genera, BrassiBase^1^, accessed on February 1, 2020) radiated into four (Franzke et al., 2011) to six (Huang et al., 2016) lineages or super-tribes ∼23 million years ago (Hohmann et al., 2015). These lineages have been divided into 52 monophyletic tribes (BrassiBase) ranging in size from the monospecific Shehbazieae (German and Friesen, 2014) to the Arabideae, which harbors more than 390 species (Jordon-Thaden et al., 2013; Karl and Koch, 2013). Many crucifer tribes do not differ in their basal or ancestral chromosome numbers. For example, tribes of lineage II/B and lineage III/E have the same number of ancestrally shared linkage groups and the same dysploid chromosomal rearrangements (Mandáková and Lysak, 2008; Mandáková et al., 2017a). By contrast, tribes of lineage I/A, such as Boechereae (*x* = 7), Descurainieae (*x* = 7), Erysimeae (mostly *x* = 7), Turritideae (*x* = 6), and Yinshanieae (*x* = 6 and 7) (Warwick and Al-Shehbaz, 2006; BrassiBase), appear to represent tribes that originated after independent reductions of the ancestral chromosome number (*n* = 8) to *n* = 7 and *n* = 6. None of the diploid Brassicaceae tribes with a clade-specific descending dysploidy have been investigated genomically, so it remains unclear whether intra-tribal diversification (i.e., speciation and origin of new genera) has involved non-dysploidal chromosomal rearrangements.

Here we focus on the tribe Boechereae which harbors c. 130 species. The vast majority of Boechereae taxa occurs only in North America, with one of these extending to Greenland and three species being endemic to the Russian Far East (Alexander et al., 2013; Doudkin and Volkova, 2013). Molecular studies (Beilstein et al., 2010; Nikolov et al., 2019) using various chloroplast and nuclear gene markers support the Boechereae (with a shared chromosome base number of *x* = 7) as a monophyletic clade sister to the New World tribe Halimolobeae (*x* = 8; 39 species in five genera, Al-Shehbaz, 2012). Alexander et al. (2013) recognized nine genera of Boechereae, seven of which (*Anelsonia* J. F. Macbride and Payson, *Cusickiella* Rollins, *Nevada* N. H. Holmgren, *Phoenicaulis* Nuttall, *Polyctenium* Greene, *Sandbergia* Greene, and *Yosemitea* P. J. Alexander and Windham) are mono- or bispecific, and, except for *Sandbergia whitedii* (Piper) Greene, restricted to the western United States. *Boechera* Á. Löve and D. Löve is by far the most diverse genus of the tribe, largely confined to the western part of the North American continent (Alexander et al., 2013). One group of eight species often assigned to *Boechera* was transferred to the genus *Borodinia* N. Busch by Alexander et al. (2013). This species group has the most discrete geographic range, apparently restricted to eastern North America and the Russian Far East [*Borodinia macrophylla* (Turcz.) O. E. Schulz]. Despite their largely allopatric distributions, *Boechera* and *Borodinia* species have hybridized in nature to produce one widespread sexual tetraploid and a series of apomictic triploids and tetraploids that erase any morphological distinctions between the two genera (Windham et al., 2014). When subsumed within *Boechera*, these lineages are informally designated the “western” and “eastern” clades, respectively.

**FIGURE 1:**
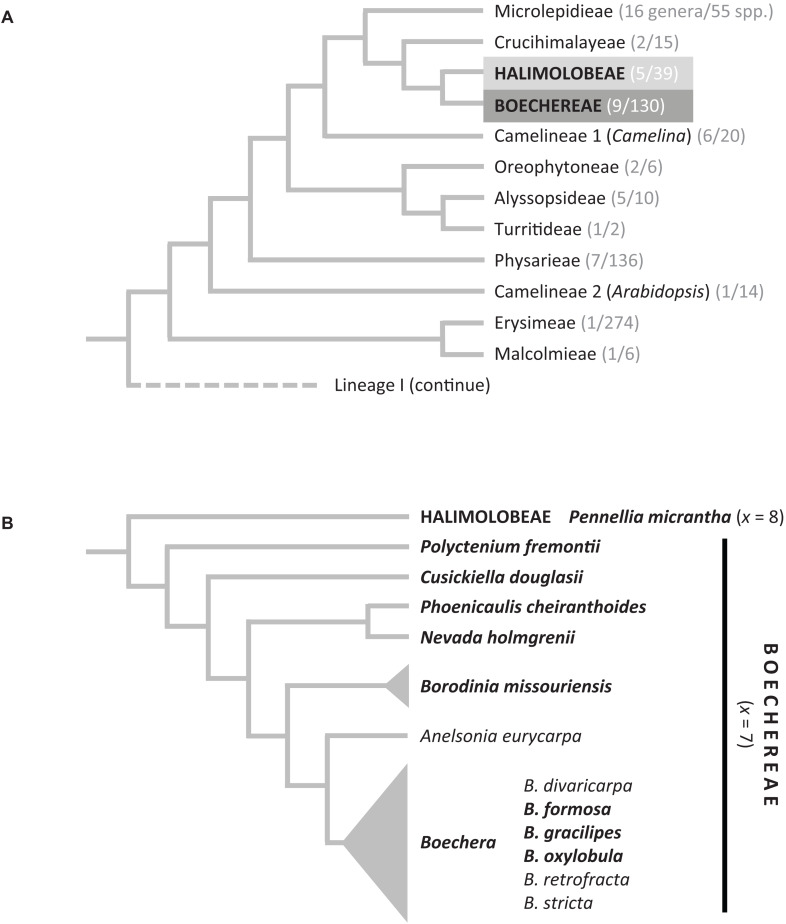
**(A)** Phylogenetic position of the Boechereae in the Brassicaceae based on Nikolov et al. (2019). In parentheses, the number of genera and species is given based on data from BrassiBase (https://brassibase.cos.uni-heidelberg.de/). **(B)** Generic relationships within the Boechereae based on Beilstein et al. (2010) (*Sandbergia* and *Yosemitea* not shown).

The species now assigned to *Boechera* (*x* = 7) were originally included in *Arabis* L. (tribe Arabideae; *x* = 8), but a series of molecular analyses (Koch et al., 2000; Beilstein et al., 2010; Nikolov et al., 2019) has shown that these genera belong to different major lineages of Brassicaceae. *Boechera* is phylogenetically closely related to the model genus *Arabidopsis* Heynh. (Figure 1A) and is best known for its classic agamic complex consisting of numerous, morphologically diverse, facultative, and obligate gametophytic apomicts. These are generally of hybrid origin, arising from a diverse array of sexual diploids with more restricted habitats. The genus is named for Danish botanist Tyge Böcher, who first documented apomixis in *Boechera holboellii* (Hornem.) Á. Löve and D. Löve (Böcher, 1951). The relatively close relationship of these species to *Arabidopsis*, combined with its diversity of ploidies (Alexander et al., 2015) and apomixis types, at both the diploid and polyploid levels (Carman et al., 2019), have made the genus a major focus for apomixis research (e.g., Naumova et al., 2001; Schranz et al., 2005; Lee et al., 2017; Kliver et al., 2018; Rojek et al., 2018; Brukhin et al., 2019). Until recently, apomixis within Boechereae was thought to be confined to the large genus *Boechera*. However, Mandáková et al. (2020) documented the occurrence of apomixis at the diploid, triploid, and tetraploid levels in one of the smaller genera of Boechereae (*Phoenicaulis*), raising the possibility that apomixis might also occur in other Boechereae genera.

In flowering plants, apomixis can be defined as asexual seed formation where clonal embryos originate either from unreduced eggs produced in unreduced female gametophytes (gametophytic apomixis) or from somatic cells of the ovule wall without an intervening unreduced gametophyte generation (sporophytic apomixis). In sporophytic apomixis, a reduced gametophyte forms, which supports the clonal embryo while it develops. The reduced gametophyte may or may not contain a sexually derived embryo (Asker and Jerling, 1992; Hand and Koltunow, 2014). Gametophytic apomixis is a prominent mode of reproduction in *Boechera* (Böcher, 1951; Roy, 1995; Naumova et al., 2001; Schranz et al., 2005), where it has greatly increased the diversity of genotypes and phenotypes by stabilizing the products of reticulate evolution (Beck et al., 2012; Carman et al., 2019). Three types of gametophytic apomixis occur in *Boechera*, and these are differentiated based on where the unreduced gametophyte forms: (*i*) if from a megasporocyte [megaspore mother cell (MMC)], it is referred to as Antennaria type diplospory, (*ii*) if from an apomeiotic dyad member of a first division meiotic restitution event, it is referred to as Taraxacum type diplospory, and (*iii*) if from a nucellar or parietal cell, it is referred to as Hieracium type apospory (Carman et al., 2019). The Antennaria type appears to be an oddity in *Boechera* that has been observed only rarely in plants that otherwise reproduce by Taraxacum type diplospory. In contrast, Taraxacum type diplospory and apospory are more commonly encountered in natural populations of *Boechera* than is sexual reproduction (Carman et al., 2019).

In the light of the fragmentary knowledge of genome evolution and reproductive modes in the Boechereae, we embarked on comparative cytogenetic and embryological analysis of several taxa representing the tribal diversity. We followed several aims: (1) To expand cytogenomic sampling of the species and genera of Boechereae to provide a more complete understanding of chromosomal evolution in the tribe, (2) to determine whether diversification within the tribe was accompanied by clade-specific chromosomal rearrangement, (3) to test whether an ancestral *n* = 7 genome inferred for *Boechera* and *Phoenicaulis* (Mandáková et al., 2015b, 2020) provides an accurate reconstruction of the ancestral genome of the tribe as a whole, and (4) to gain insights into the reproductive modes of previously unsampled Boechereae genera by conducting embryological analyses on most of the species analyzed cytogenomically.

## Materials and Methods

### Species Analyzed

Ten Boechereae species were selected to span the phylogenetic diversity of the tribe (Figure 1). These included *Boechera formosa* (Greene) Windham and Al-Shehbaz (2*n* = 14), *Boechera gracilipes* (Greene) Dorn (2*n* = 14), *Boechera oxylobula* (Greene) W. A. Weber (2*n* = 14), *Borodinia missouriensis* (Greene) P. J. Alexander and Windham (2*n* = 14), *Cusickiella douglasii* (A. Gray) Rollins (2*n* = 28), *Nevada holmgrenii* (Rollins) N. H. Holmgren (2*n* = 14), *Phoenicaulis cheiranthoides* (2*n* = 14, 21, 28), *Polyctenium fremontii* (S. Watson) Greene (2*n* = 28), *Sandbergia perplexa* (L. F. Henderson) Al-Shehbaz (2*n* = 14), and *S. whitedii* (2*n* = 21). As the tribe Halimolobeae was repeatedly retrieved as a sister clade to Boechereae (e.g., Beilstein et al., 2010; Couvreur et al., 2010; Alexander et al., 2013), *Pennellia micrantha* (A. Gray) Nieuwland (2*n* = 16) was selected to represent the Halimolobeae clade outgroup. The origins of the analyzed populations are listed in Supplementary Table S1; multiple individuals were analyzed from each population.

The analyzed plants were either collected in the wild or grown in a growth chamber from seeds collected in the wild. Young inflorescences of the analyzed plants were collected and fixed in freshly prepared fixative (ethanol: acetic acid, 3: 1) overnight, transferred to 70% ethanol and stored at −20°C.

### Chromosome Preparation

Chromosome spreads from fixed young flower buds containing immature anthers were prepared according to published protocols (Lysak and Mandáková, 2013; Mandáková and Lysak, 2016a). Chromosome preparations were treated with 100 μg/ml RNase in 2 × sodium saline citrate (SSC; 20 × SSC: 3 M sodium chloride, 300 mM trisodium citrate, pH 7.0) for 60 min and with 0.1 mg/ml pepsin in 0.01 M HCl at 37°C for 5 min, then post-fixed in 4% formaldehyde in distilled water, and dehydrated in an ethanol series (70, 90, and 100%, 2 min each).

### DNA Probes

The BAC clone T15P10 (AF167571) of *Arabidopsis thaliana* (L.) Heynh. bearing 35S rRNA gene repeats was used for *in situ* localization of nucleolar organizer regions (NORs), and the *A. thaliana* clone pCT4.2 (M65137), corresponding to a 500 bp 5S rDNA repeat, was used for localization of 5S rDNA loci. For Comparative Chromosome Painting (CCP), 674 chromosome-specific BAC clones of *A. thaliana* (The Arabidopsis Information Resource, TAIR^2^) were used to establish contigs corresponding to the 22 genomic blocks (GBs) and eight chromosomes (AK1–AK8) of the Ancestral Crucifer Karyotype (ACK; Lysak et al., 2016). See Supplementary Tables S2–S8 for the list of BAC clones used to identify the 22 GBs on chromosomes of the Boechereae species. To determine and characterize inversions and split GBs, some BAC contigs were split into smaller subcontigs and differentially labeled (e.g., Aa, Ab, Ca, Cb, see Supplementary Tables S3–S8). All DNA probes were labeled with home-made biotin-dUTP, digoxigenin-dUTP, or Cy3-dUTP by nick translation as described by Mandáková and Lysak (2016b).

### Comparative Chromosome Painting (CCP)

DNA probes were pooled to follow the design of a given experiment, ethanol precipitated, dried, and dissolved in 20 μl of 50% formamide and 10% dextran sulfate in 2 × SSC. The 20 μl of the dissolved probe were pipetted on a chromosome-containing slide and immediately denatured on a hot plate at 80°C for 2 min. Hybridization was carried out in a moist chamber at 37°C overnight. Post-hybridization washing was performed in 20% formamide in 2 × SSC at 42°C three times (5 min each time). Hybridized probes were visualized either as the direct fluorescence of Cy3-dUTP or through fluorescently labeled antibodies against biotin-dUTP and digoxigenin-dUTP following Mandáková and Lysak (2016b). Chromosomes were counterstained with 4′,6-diamidino-2-phenylindole (DAPI, 2 μg/ml) in Vectashield antifade. Fluorescence signals were analyzed and photographed using a Zeiss Axioimager epifluorescence microscope with a CoolCube camera (MetaSystems). Images were acquired separately for all four fluorochromes using appropriate excitation and emission filters (AHF Analysentechnik). The four monochromatic images were pseudocoloured, merged, and cropped using Photoshop CS (Adobe Systems) and ImageJ (National Institutes of Health).

### *In silico* Sequence Analysis

*Boechera stricta* (Graham) Al-Shehbaz (v1.2; Lee et al., 2017), *Arabidopsis lyrata* (L.) O’Kane and Al-Shehbaz (v2.1; Hu et al., 2011), and *A. thaliana* (TAIR 10) genome assemblies and annotations were downloaded from the Phytozome webpage^3^. Inter-genome collinearity was analyzed by SynOrths, identifying whether two homeologous genes are a conserved syntenic pair based on their sequence similarity and the support of homeologous flanking genes (Cheng et al., 2012).

### Embryological Analyses

Clusters of pre-anthesis staged floral buds were fixed in 3:1 fixative for 48 h and stored in 70% EtOH. Ovaries were excised, cleared, measured, and mounted following Mandáková et al. (2020). An Olympus (Center Valley, PA, United States) BX53 microscope with differential interference contrast (DIC) optics and equipped with a DP74 digital camera with cellSens Dimension 1 software (Olympus) was used to investigate parietal cell, MMC, and female gametophyte origins.

## Results

**FIGURE 2:**
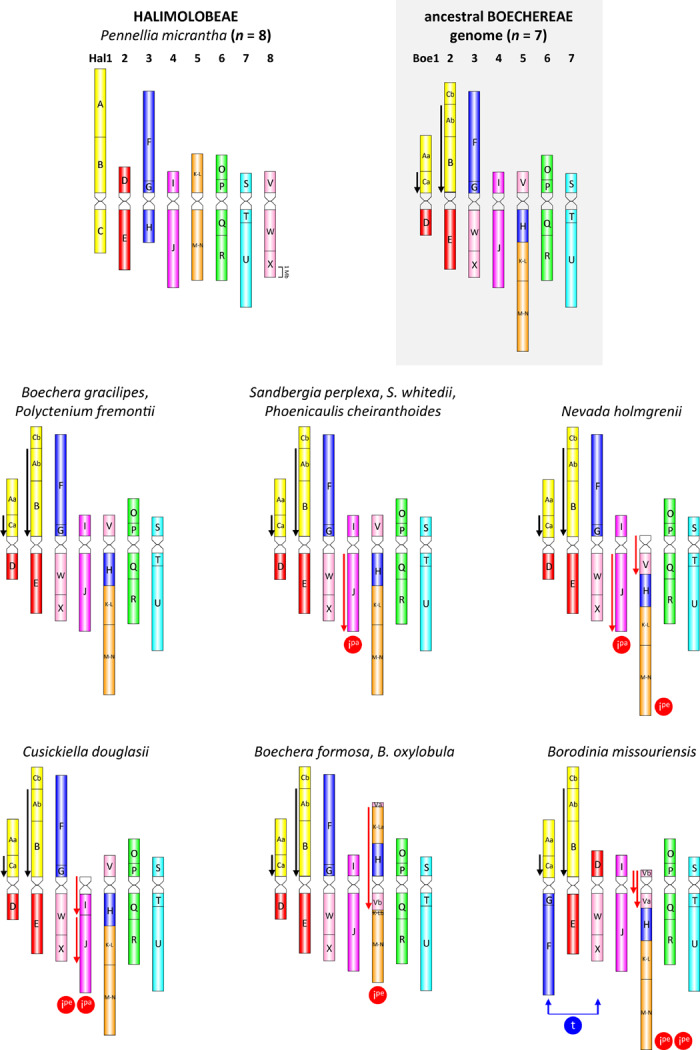
Comparative cytogenomic maps of *Pennellia micrantha* (Halimolobeae), the ancestral Boechereae genome, and 10 analyzed Boechereae species. As subgenomes of the polyploid species/cytotypes have the same structure, only a single (sub)genome is shown for triploids (*P. cheiranthoides*, *S. whitedii*) and tetraploids (*C. douglasii*, *P. cheiranthoides*, *P. fremontii*). Color coding of 22 genomic blocks (A to X) reflects their position on the eight ancestral chromosomes (AK1–AK8) in the Ancestral Crucifer Karyotype (Lysak et al., 2016). Blocks split into two segments are labeled as “a” and “b.” Downward-pointing arrows denote the inverse orientation of GBs compared to their position in the ACK represented here by the *P. micrantha* genome. Black arrows mark the Boechereae-specific inversions which occurred prior to the divergence of the tribe, whereas red arrows denote genus- and species-specific inversions that occurred after the divergence of Boechereae. I^*pe*^: pericentric inversion; I^*pa*^: paracentric inversion; t: whole-arm translocation. All ideograms are drawn to scale, whereby the size of GBs corresponds to the size of homeologous blocks in the *Arabidopsis thaliana* genome (The Arabidopsis Information Resource, TAIR; http://www.arabidopsis.org). Genome structure of *P. cheiranthoides* was adopted from Mandáková et al. (2020).

Based on the ACK, and on previously analyzed Boechereae species (Mandáková et al., 2015a, 2020), detailed comparative cytogenetic maps were constructed by CCP for each of the 10 Boechereae species and for *P. micrantha* (Figure 2 and Supplementary Tables S2–S8).

### The Outgroup *Pennellia micrantha* Genome Structurally Mirrors the ACK

**FIGURE 3:**
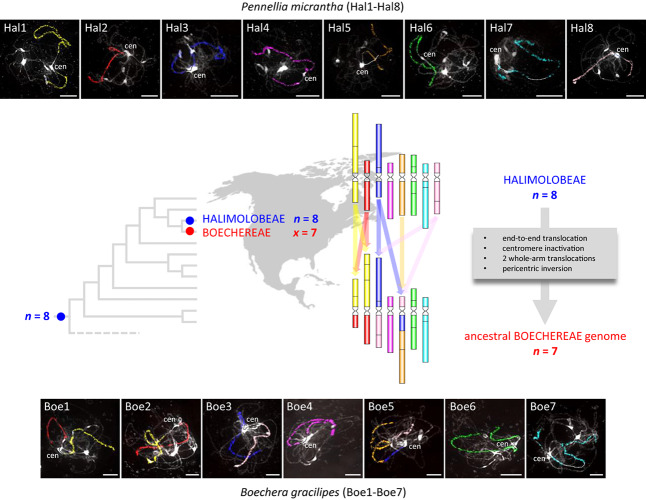
The inferred origin of the ancestral Boechereae genome and painted chromosomes of *Pennellia micrantha* (Hal1–Hal8) and *Boechera gracilipes* (Boe1–Boe7). The ancestral Boechereae genome (*n* = 7) originated from a Halimolobeae-like genome (*n* = 8) through descending dysploidy mediated by an end-to-end translocation accompanied by a centromere inactivaton, two additional reciprocal translocations and a pericentric inversion (see Figure 4 for more details). In the CCP images, different colors correspond to the eight ancestral chromosomes (AK1–AK8) in the Ancestral Crucifer Karyotype (Lysak et al., 2016), whereas capital letters refer to 22 genomic blocks (A to X). *A. thaliana* BAC clones defining each BAC contig/painting probe in *Pennellia* and *Boechera* are listed in Supplementary Tables S2, S3, respectively. Chromosomes were counterstained by DAPI. The fluorescence signals of the painting probes were captured as black and white photographs, and the signals were then pseudocolored to match the eight chromosomes of the ACK. Scale bars, 10 μm.

Comparative chromosome painting in *P. micrantha* (2*n* = 16, Halimolobeae) was successful in identifying all 22 conserved GBs making up the eight chromosomes (Hal1–Hal8, Figures 2, 3 and Supplementary Table S2). The ACK-like *Pennellia* genome further corroborrated the earlier assumption (Mandáková et al., 2015b, 2020) that the Most Recent Common Ancestor (MRCA) of Boechereae and Halimolobeae had eight chromosomes and structurally resembled the ancestral genome of crucifer lineage I.

### Overall Structural Stasis of Boechereae Genomes

Comparative chromosome painting with painting probes designed according to the structure of the seven linkage groups in *Boechera* (Mandáková et al., 2015a) and *Phoenicaulis* (Mandáková et al., 2020), were effective in identifying all seven or 14 chromosome pairs among the 10 Boechereae species analyzed. All 10 genomes had a very similar organization (Figure 2), except for a few species-specific chromosomal rearrangements (see below). The overall structural genome similarity among different Boechereae genera allowed us to reconstruct the genome of the MRCA of Boechereae.

### Ancestral Boechereae Genome

**FIGURE 4:**
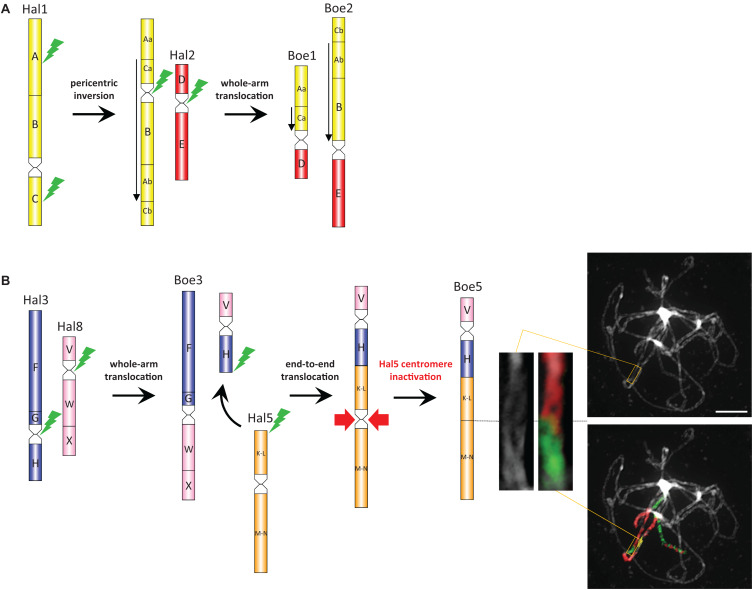
The origin of Boechereae-specific chromosomes. Parsimoniously reconstructed origin of the ancestral Boechereae chromosomes Boe1, Boe2 **(A)**, Boe3, and Boe5 **(B)** from the ACK/Halimolobeae-like chromosomes Hal1–Hal3, Hal5, and Hal8. Chromosome images show painting of the Boe5 homeolog at pachytene in *Boechera gracilipes* including the K-L/M-N region with the inactive AK5 paleocentromere (magnified inset). Downward-pointing arrows refer to GBs inverted compared to their orientation in the ACK/Halimolobeae genome. Capital letters mark the ancestral GBs of the ACK (Lysak et al., 2016). Green flash-like symbols indicate chromosome breakpoints; red arrows indicate the lost of Hal5 (AK5) centromere on Boe5. Scale bar, 10 μm.

By comparing the 10 Boechereae genomes studied herein with those of three diploid *Boechera* taxa (Mandáková et al., 2015b), *Phoenicaulis* (Mandáková et al., 2020), and *P. micrantha*, we inferred the ancestral Boechereae genome with seven pairs of chromosomes (Boe1–Boe7, Figures 2, 3). Three of these pairs (Boe4, Boe6, and Boe7) retained their ancestral structure as in the ACK, or Halimolobeae, whereas four pairs (Boe1–Boe3 and Boe5) are specific to the Boechereae genomes (Figures 2–4 and Supplementary Table S3).

CCP chromosome painting analyses allowed us to reconstruct the origin of the four Boechereae-specific chromosomes. The origin of chromosomes Boe1 and Boe2 (Figure 4A) most likely included an initial 0.52-Mb pericentric inversion on ancestral chromosome AK1 with breakpoints within GBs A [between BAC clone F13B4 (At1g13620) and T16N11 (At1g15410)] and C [between F8L10 (At1g53170) and F12M16 (At1g53160)]. The size of this inversion and other documented rearrangements were inferred from the physical length (Mb) of *A. thaliana* BAC contigs spanning these chromosome regions. The inversion-bearing AK1 chromosome underwent a whole-arm translocation with paleochromosome AK2 resulting in chromosomes Boe1 (GBs Aa, Ca, and D) and Boe2 (Cb, Ab, B, and E). Chromosome Boe3 (F, G, W, and X) originated by a whole-arm translocation between paleochromosomes AK3 and AK8 (Figure 4B). The second translocation chromosome (GBs V and H) was involved in an end-to-end translocation with chromosome AK5, mediating the chromosome number reduction (8 → 7) in Boechereae. The collinearity of GBs K-L and M-N and the absence of the original centromere suggest that the “chromosome fusion” was accompanied or followed by a removal of the AK5 paleocentromere (Figure 4B). Remnants of the AK5 paleocentromere, apparent as heterochromatic knobs and/or unpainted chromosome segments, were not observed in any of the analyzed species (Figures 3, 4B).

### Clade-Specific Chromosomal Rearrangements

**FIGURE 5:**
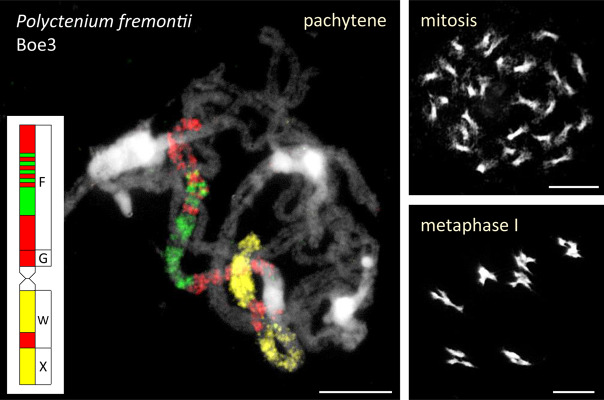
Quadrivalents in *Polyctenium fremontii* (2*n* = 4*x* = 28). The 28 chromosomes form seven quadrivalents during the first meiotic division. The quadrivalent of Boe3 homeologs was identified by CCP of pachytene chromosomes with BAC contigs specific for genomic blocks F, G, W, and X (Supplementary Table S3). Chromosomes were counterstained by DAPI. Scale bars, 10 μm.

The ancestral Boechereae genome remained conserved in the diploid *B. gracilipes* (Figures 2, 3 and Supplementary Table S3) and the tetraploid *P. fremontii* (Figure 2 and Supplementary Table S3). Given that both *Polyctenium* subgenomes had identical chromosome structure and the pachytene chromosomes formed quadrivalents (Figure 5), the analyzed accession of *P. fremontii* was most likely of autotetraploid origin.

**FIGURE 6:**
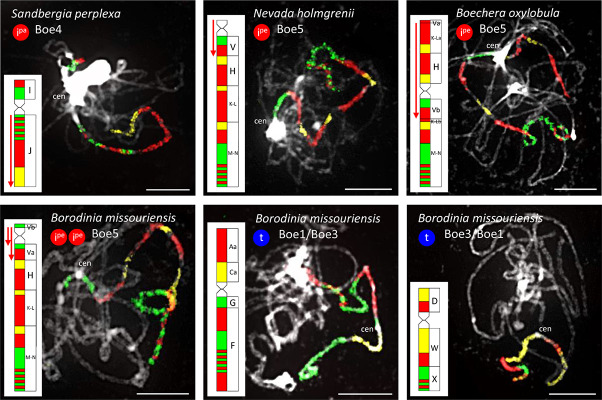
Species- and genus-specific chromosomal rearrangements in diploid Boechereae revealed by CCP. Red downward-pointing arrows denote inverse orientation of GBs compared to their position in the ancestral Boechereae genome (Figure 3). I^*pe*^: pericentric inversion; I^*pa*^: paracentric inversion; t: whole-arm translocation. Chromosomes were identified by CCP with *A. thaliana* BAC contigs labeled by biotin-dUTP (red flourescence), digoxigenin-dUTP (green) and Cy3-dUTP (yellow). *A. thaliana* BAC clones defining each BAC contig/painting probe are listed in Supplementary Tables S4, S5, S7, S8. Chromosomes were counterstained by DAPI. Scale bars, 10 μm.

In the three cytotypes of *P. cheiranthoides* (2*x*, 3*x*, and 4*x*; Mandáková et al., 2020), diploid *N. holmgrenii*, diploid *S. perplexa*, and triploid *S. whitedii*, chromosome Boe4 was altered by an 8.24-Mb paracentric inversion spanning the entire block J (whole long arm). The breakpoints were located in the pericentromeric and subtelomeric regions (Figures 2, 6 and Supplementary Tables S4, S5). The absence of subgenome differentiation in the analyzed triploid and tetraploid populations of *Phoenicaulis* and *S. whitedii* suggests intra-specific, autopolyploid origins for these polyploids. Additionally, *N. holmgrenii* exhibited a 2.55-Mb whole-arm pericentric inversion on Boe5. In this population, the short arm (GB V) was inverted, rendering the acrocentric chromosome telocentric (Figures 2, 6 and Supplementary Table S5).

Chromosome Boe4 of tetraploid *C. douglasii* also displayed inversions (Figure 2 and Supplementary Table S6). A 2.06-Mb pericentric inversion, spanning the whole short arm (GB I), converted the chromosome from acrocentric to telocentric. Boe4 was also modified by a 5.35-Mb paracentric inversion [breakpoint between blocks I and J, and within block J—between T28M21 (At2g40090) and T3G21 (At2g40240)]. The *Cusickiella* population analyzed for this study was most likely of autotetraploid origin given that both subgenomes were structurally similar, including a reshuffling of Boe4 (data not shown). Finally, in *B. formosa* and *B. oxylobula*, Boe5 was altered by a 9.83-Mb pericentric inversion with breakpoints within block V [between K14A3 (At5g47175) and MQL5 (At5g47150)] and K-L [between MQP15 (At3g30655) and MED5 (At3g30663)], converting the chromosome from acrocentric to metacentric (Figures 2, 6 and Supplementary Table S7).

*Borodinia missouriensis* had the most reshuffled genome encountered among the taxa analyzed (Figures 2, 6 and Supplementary Table S8). A whole-arm translocation between Boe1 (GBs Aa, Ca, and D) and Boe3 (F, G, W, and X) produced two *B. missouriensis*-specific translocation chromosomes. The species also shared a 2.55-Mb whole-arm pericentric inversion spanning block V of Boe5 with *N. holmgrenii*. In *B. missouriensis*, this was followed by a small 0.57-Mb pericentric inversion splitting block V into Va and Vb and placing the Boe5 centromere close to the chromosome terminus between BACs K24F5 (At5g43211) and MNL12 (At5g43190).

### Localization of rDNA Loci

In *P. micrantha*, NORs (35S rDNA loci) were localized on the termini of five chromosomes (Hal1, Hal3, Hal4, Hal6, and Hal7) and two 5S rDNA loci were adjacent to the pericentromeric heterochromatin of chromosomes Hal4 and Hal6 (Supplementary Figure S1). A single NOR and 5S rDNA locus were identified in eight Boechereae species analyzed; two NORs were found in *Cusickiella* and two 5S loci in *Nevada* (Supplementary Figure S1). NORs were located terminally on the short chromosome arm of Boe4 (*Boechera* spp. and *Cusickiella*) or Boe5 (*Borodinia*, *Cusickiella*, *Polyctenium*, and *Nevada*). In *Phoenicaulis* and both *Sandbergia* species, NORs were located interstitially, close to the pericentromere of Boe6. This supports the close relationship between *Phoenicaulis* and *Sandbergia*. Interestingly, all telocentric chromosomes were NOR-bearing (Boe4 in *Cusickiella* and Boe5 in *Borodinia* and *Nevada*). 5S rDNA loci were found positioned interstitially, close to the pericentromere of Boe5 (*Cusickiella*, *Nevada*, and *Phoenicaulis*), Boe6 (*Boechera* spp. and *Polyctenium*), and Boe7 (*Borodinia*, *Nevada*, and both *Sandbergia* spp.).

In triploid (*Phoenicaulis*, *S. whitedii*) and tetraploid (*Cusickiella*, *Phoenicaulis*, *Polyctenium*) taxa/cytotypes, the position of rDNA gene loci at the same chromosomal positions within three (triploid) or four (tetraploid) chromosome sets, further supported the purported autopolyploid origins of these genomes.

### The Inactive Centromere Between Genomic Blocks K-L and M-N in *Boechera stricta*

**FIGURE 7:**
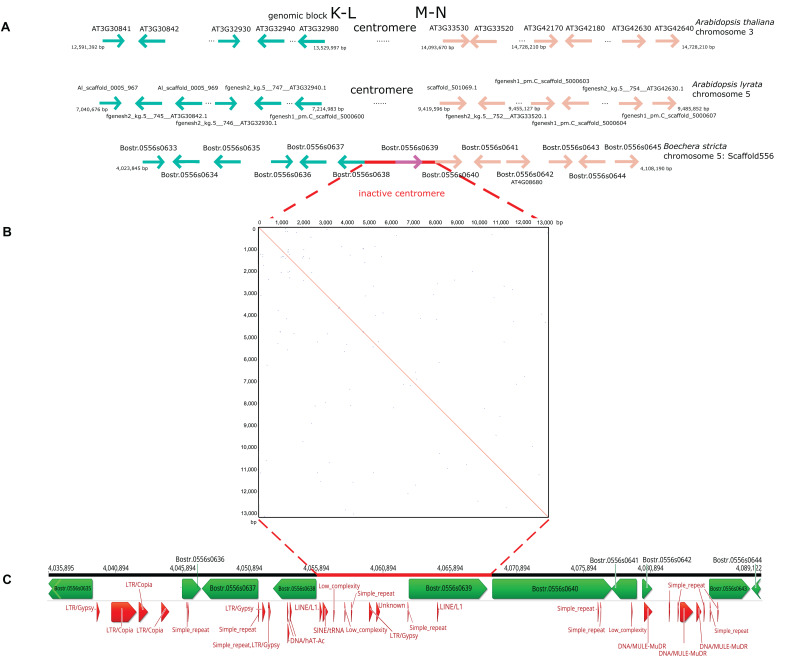
Sequence comparison of the AK5 centromeric region on homeologous chromosomes in *Arabidopsis thaliana*, *A. lyrata* and *Boechera stricta*. **(A)** The functional centromere on chromosome 3 and 5 in *A. thaliana* and *A. lyrata*, respectively, is located between genomic blocks K-L and M-N, between At3g32980 and At3g33530. The 13-kb region corresponding to the inactive AK5 centromere is located between genes Bostr.0556s0638 and Bostr.0556s0640 on chromosome Boe5. **(B)** A dot-plot self-comparison of the 13-kb region on Boe5 showing the absence of tandem repeat arrays. **(C)** Annotation of the *B. stricta* scaffold 556 including the 13-kb region and regions 20 kb upstream and downstream. Transposable elements and their remnants do not exhibit a higher abundance within the 13-kb region.

The *B. stricta* genome contains an inactive centromere between GBs K-L and M-N on chromosome Boe5. To characterize this region at the sequence level, we compared scaffold 556 in the *B. stricta* assembly with orthologous genes on homeologous chromosome 3 and 5 in *A. thaliana* and *A. lyrata*, respectively (Figure 7A). The centromeric region is delimited by genes of the Peroxidase superfamily (loci At3g32980) and genes of the Transducin family (loci At3g33530). In *B. stricta*, the site of the eliminated (AK5) paleocentromere corresponds to a 13-kb region between orthologs Bostr.0556s0638 and Bostr.0556s0640. This region contains a single gene (Bostr.0556s0639), which is presumably paralogous to gene Bostr.13158s0074 (homology of 84.9%), located in the distant part of block M-N on the same chromosome. In the *Arabidopsis* genomes, orthologs of Bostr.13158s0074 are located within M-N on chromosomes At3 (At3g60740; At3: 22,447,245-22,453,364) and Al5 (scaffold_503309.1; Al5: 20,028,781−20,034,751), respectively. Additionally, the *A. lyrata* genome possesses a paralog, Al_scaffold_0002_1021, located on Al2 in GB E (position 9,412,104-9,422,712). No remnants of tandem repeats were detected within the 13-kb region (Figure 7B). Comparable distribution of transposable elements and their remnants was observed within the former centromeric region, in upstream and downstream 20 kb regions (Figure 7C) and along the whole Boe5 pseudo-chromosome. The absence of repeat segment enrichment within the short 13-kb region supports an almost complete removal of the AK5 paleocentromere after the end-to-end “chromosome fusion” (Figure 4B).

### The Heterochromatic *Het* Chromosome Was Absent in the Analyzed Boechereae Species

In apomictic *Boechera* species (2*n* = 14) and *Phoenicaulis* cytotypes (2*n* = 21 and 28, but not apomictic 2*n* = 14), one of the Boe1 homologs (a *Het* chromosome) displayed expanded regions of pericentromeric heterochromatin (Mandáková et al., 2015a, 2020). In all analyzed species of the present study, pericentromeric heterochromatin regions of the Boe1 homologs were comparable in size.

### High Frequency Apospory Occurs in *Polyctenium* and *Sandbergia*

**FIGURE 8:**
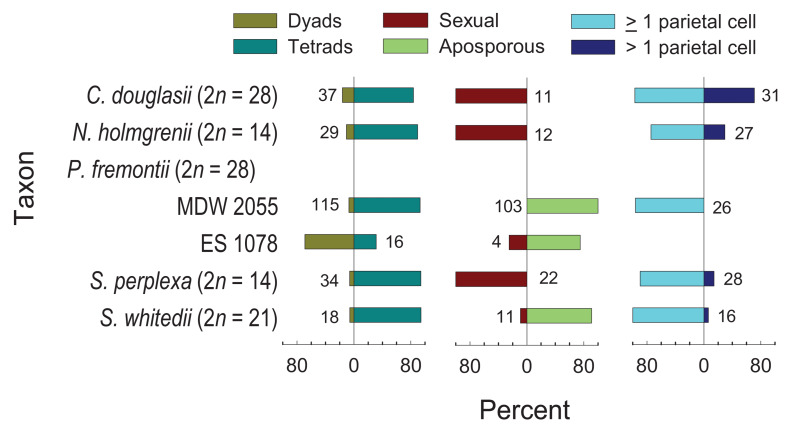
Frequencies of dyads vs. tetrads, sexual vs. aposporous gametophytes, and parietal cells or tissues (≥1 parietal cell) vs. parietal tissues (>1 parietal cell). Numbers next to bars represent observations contributing to each variable pair.

Six accessions (Supplementary Table S1), representing five species distributed across four genera, were embryologically analyzed (Figure 8). Tetraploid *C. douglasii*, diploid *N. holmgrenii*, and diploid *S. perplexa*, were sexual. Tetraploid *P. fremontii* (MDW 2055, ES 1078) and triploid *S. whitedii* were highly aposporous. In the *P. fremontii* population ES 1078, the dyad to tetrad ratio was high (Figure 8), which in *Boechera* would generally indicate diplospory (Carman et al., 2019). However, three of the four observed gametophytes were forming from nucellar cells (apospory), and the fourth was forming from the surviving megaspore of a sexual tetrad. While this sample size is too small to rule out diplospory, our observations indicated that apospory initiates early during ovule development with meiosis regularly terminating as early as the sexual dyad to early tetrad stages. Such termination may have inflated the dyad to tetrad ratio (Figure 8).

**FIGURE 9:**
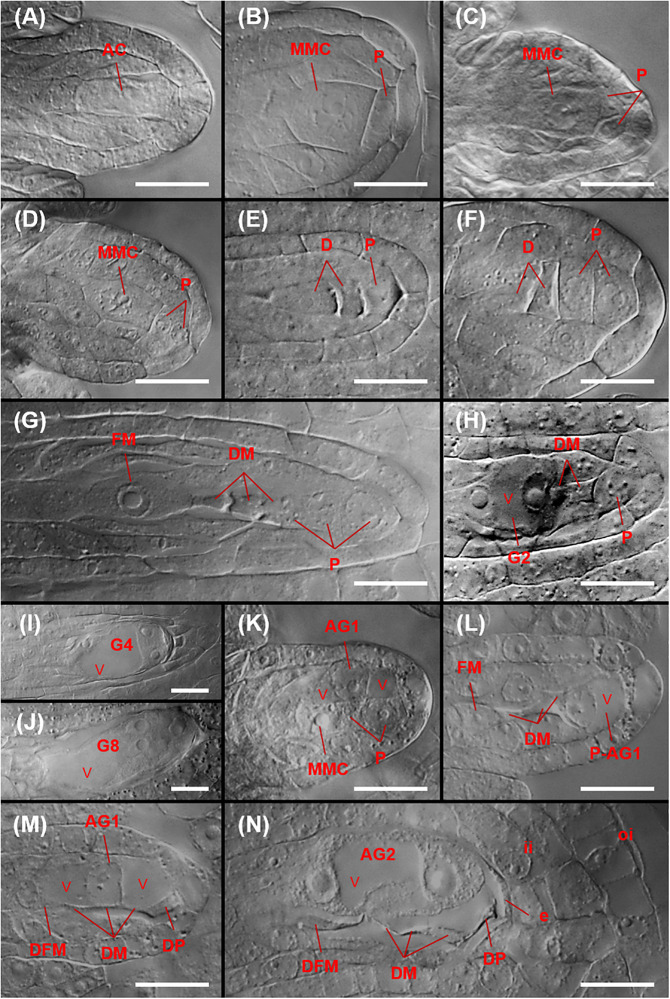
Megasporogenesis and sexual and aposporous gametophyte formation in *P. fremontii* **(A,K–N)** and sexual *C. douglasii* **(B,D,F,G)**, *N. holmgrenii* **(C)**, and *S. perplexa* **(E,H–J)**. **(A)** Archesporial cell (AC) at the budding integument stage. **(B)** Mitotic division of an AC yielding a proximal MMC and a distal parietal cell (P). **(C,D)** Anticlinal and paraclinal divisions, respectively, of a P to yield a two-celled parietal tissue. **(E,F)** Dyads (D) with one and two Ps, respectively. **(G)** Tetrad showing the functional megaspore (FM) and three degenerating megaspores (DM). Also shown is a parietal tissue consisting of three Ps that formed from two paraclinal divisions of the original P. **(H)** Two-nucleate sexual gametophyte (G2) showing a central vacuole (v), two of three DM, and a P. **(I)** Four-nucleate sexual gametophyte (G4) with three nuclei visible. **(J)** Eight-nucleate sexual gametophyte (G8) showing egg apparatus formation at the micropylar end of the gametophyte. **(K)** 1-nucleate aposporous gametophyte (AG1) from a nucellar cell at the MMC stage. **(L)** AG1 from a parietal cell (P-AG1) at the tetrad stage. **(M)** AG1 from a nucellar cell at the late tetrad stage showing functional megaspore degeneration (DFM), DMs, degenerating nucellar cells, and a degenerating parietal cell (DP). **(N)** Two-nucleate aposporous gametophyte (AG2) at the late tetrad stage showing a DFM, DMs, degenerating nucellar cells, a degenerating parietal cell (DP), and degenerating epidermal cells. Scale bars, 20 μm.

Parietal cell frequencies were ≥ 70% for five of the six taxa studied (Figure 8). These frequencies are similar to those observed in *Phoenicaulis* (Mandáková et al., 2020), but they are much higher than those generally observed in *Boechera* (<50%) (Naumova et al., 2001; Carman et al., 2019). *The ES 1078 P. fremontii* sample was too small to determine this frequency. Parietal cells form from the distal daughter cell of the mitotic division of the archesporial cell. In these cases, the MMC forms from the proximal daughter cell (Figures 9A,B). In ovules of tenuinucellate species, the opposite normally occurs, i.e., the MMC differentiates distally, and the proximal cell is considered nucellar (Johri et al., 1992). In this respect, the Boechereae show tendencies toward crassinucellate development, with parietal cells sometimes undergoing further division to produce a parietal tissue that positions the meiocyte deeper within the ovule (Figures 9C–G).

Sexual and aposporous gametophyte formation were of the eight-nucleate Polygonum type (Figures 9H–J). In the aposporous taxa, one or more nucellar cells, and sometimes parietal cells, initiated vacuolate gametophyte formation as early as MMC differentiation (Figures 9K,L). As described above, this may have terminated meiosis prior to M_*II*_ and caused the abnormally high sexual dyad to tetrad ratio observed for the *P. fremontii* population ES 1078 (Figure 8). Nucellar, parietal, and nucellar epidermis cells degenerated quickly in front of the rapidly growing sexual or aposporous gametophytes (Figures 9H–N).

## Discussion

### The Ancestor of the Boechereae Had Seven Chromosomes and Descended From an ACK-Like Genome With Eight Chromosomes

Using BAC-based CCP, we analyzed 14 samples of Boechereae representing seven of the nine genera recognized by Alexander et al. (2013). These analyses revealed a high level of genomic stasis across the tribe, with the earliest diverging genus *Polyctenium* (Figure 1; Beilstein et al., 2010; Couvreur et al., 2010) and *B. gracilipes* (a member of the derived “western *Boechera* clade,” Figure 1) showing identical chromosome structures (Figure 2). Our analysis corroborrated our earlier hypothesis (Mandáková et al., 2015b, 2020) of an ancestral Boechereae genome with seven chromosomes (*n* = 7) derived from an older *n* = 8 genome by descending dysploidy. This precursor *n* = 8 genome structurally resembled the ACK, an ancestral genomic arrangement present in many tribes of crucifer lineage I (Lysak et al., 2016). Indeed, analysis of the *P. micrantha* genome from the Halimolobeae, sister group to the Boechereae, confirmed that the eight chromosomes of Halimolobeae genomes (Bailey et al., 2007) are homeologous to the eight chromosomes of the ACK. While the eight ancestral chromosomes remained conserved in Halimolobeae, the divergence of extant Boechereae appears to coincide with or follow a descending dysploidal change from *n* = 8 to *n* = 7.

### Tribe-Specific Descending Dysploidy

Given that all sampled Boechereae exhibit a chromosome base number of *x* = 7, it is likely that the descending dysploidy event documented above occurred prior to the initial divergence of the extant members of the tribe approximately 8 million years ago (Couvreur et al., 2010). This reduction of chromosome number did not result from a “simple fusion” of two chromosomes. The first step involved the origin of a transient translocation (AK3/AK8) chromosome by a whole-arm translocation, followed by an end-to-end translocation between AK3/AK8 and AK5 (→ chromosomes Boe3, Boe5). The chromosome-arm collinearity of the resulting Boe5 points to inactivation or loss of the AK5 centromere. Interestingly, this paleocentromere has disappeared from many other crucifer genomes independently, and it is the most frequently inactivated centromere detected so far among the tribes of lineage I (*Camelineae*: Lysak et al., 2016; *Cardamineae*: Mandáková et al., 2016; *Microlepidieae*, Mandáková et al., 2010, 2017b). As tribal divergence post-dates the origin of four tribe-specific chromosomes, we propose that the three translocations involving five out of eight ancestral chromosomes were a key evolutionary innovation underlying the origin and diversification of the Boechereae in North America.

### Genus- and Species-Specific Inversions

Although reciprocal translocations clearly played a major role in the origin of the tribe, the only additional translocation noted within the tribe was a unique whole-arm transfer between Boe1 and Boe3 that produced two structurally unique chromosomes in *B. missouriensis* (Figure 2). However, chromosomal inversions proved to be common within the group, as they are in other mustards (e.g., Mandáková et al., 2015a; Fransz et al., 2016) and land plants in general (Hoffmann and Rieseberg, 2008). Autapomorphic pericentric inversions on chromosome Boe5 were observed in both *Borodinia* and *Nevada*, while *Cusickiella* exhibited unique pericentric and paracentric inversions on Boe4 (Figure 2).

Our analyses identified three potentially synapomorphic chromosomal rearrangements within the tribe: (1) a 9.83-Mb pericentric inversion on Boe5 shared by *B. formosa* and *B. oxylobula*, but not by *B. gracilipes*, (2) a 2.55-Mb pericentric inversion on Boe5 shared by *N. holmgrenii* and *Borodinia missouriensis*, and (3) a 8.24-Mb paracentric inversion on Boe4 shared by *N. holmgrenii*, all three ploidies of *P. cheiranthoides*, and both species of *Sandbergia* (Figure 2). Support for the synapomorphic status of these three chromosomal rearrangements is equivocal in the few phylogenetic analyses of Boechereae published to date.

With respect to the 9.83-Mb pericentric inversion apparently shared by *B. formosa* and *B. oxylobula*, the only phylogenetic analysis with appropriate taxon sampling is the concatenated nuclear gene tree of Alexander et al. (2013). This tree shows very weak support for a clade encompassing *B. oxylobula* and *B. gracilipes* but excluding *B. formosa.* A close relationship between *B. oxylobula* (which has the inversion) and *B. gracilipes* (which does not) is congruent with morphology in this case, and it argues against the 9.83-Mb pericentric inversion being synapomorphic.

Concerning the 2.55-Mb pericentric inversion shared by *N. holmgrenii* and *B*. *missouriensis*, the two published analyses with appropriate taxon sampling yielded conflicting topologies. The concatenated nuclear gene tree of Alexander et al. (2013) provides very weak support for a sister relationship between *Nevada* and *Borodinia*, which would favor the interpretation of the 2.55-Mb inversion as a synapomorphic character. However, the Boechereae phylogeny presented by Beilstein et al. (2010) places *Nevada* as sister to *Phoenicaulis* not *Borodinia*, the latter being sister to a clade comprising *Boechera s.s.* and *Anelsonia* (Figure 1B). If this phylogeny is correct, the 2.55-Mb inversion apparently shared by *Nevada* and *Borodinia* would have originated independently.

The final potential chromosomal synapomorphy to be considered is the 8.24-Mb paracentric inversion shared by *N. holmgrenii*, all three ploidies of *P. cheiranthoides*, and both species of *Sandbergia* (Figure 2). Among previously published phylogenies, the Beilstein et al. (2010) topology hypothesizes a sister relationship between *Nevada* and *Phoenicaulis* (*Sandbergia* was not sampled). This would be congruent with the 8.24-Mb inversion on Boe4 being interpreted as synapomorphic. However, the concatenated nuclear gene tree of Alexander et al. (2013) hypothesizes a sister relationship (very weakly supported) between *Nevada* and *Borodinia* and provides no resolution of inter-generic relationships for *Phoenicaulis* or *Sandbergia*. This creates a conflict between the only two inversions that could be a synapomorphy. If the Beilstein et al. (2010) phylogeny is correct, then the 8.24-Mb inversion discussed here could be synapomorphic but the 2.55-Mb inversion mentioned previously would not. On the other hand, if the Alexander et al. (2013) topology is correct, then the 2.55-Mb inversion could be synapomorphic but the 8.24-Mb inversion would not. A better resolved and supported phylogeny will be needed to assess the homology of the chromosomal rearrangements documented herein.

Chromosomal inversions appear to be relatively common in Boechereae, but the existence of breakage “hotspots” on several chromosomes can make it difficult to infer homology. In our dataset, most of the inversions detected are pericentric and occur in just one or two samples or taxa. The only inversion that appears to have any time depth is the 8.24-Mb paracentric inversion on Boe4 (shared by six samples representing four species and three genera). The others appear to be more recent, like the 8.4-Mb paracentric inversion on chromosome Bs1 that distinguishes the West genotype of *B. stricta* from other populations of the species (Lee et al., 2017). Some of these young inversions likely originated since the last glacial maximum, suggesting that this type of chromosomal rearrangement may be an ongoing and important contributor to reproductive isolation and speciation within the Boechereae.

### The Boechereae *n* = 8 Ancestor in a Phylogeographic Context

Eurasia, specifically the Irano-Turanian floristic region, is believed to be the cradle of crucifer origin (Franzke et al., 2009). Although long-distance dispersal events contributed to plant migrations from Eurasia to North America and in the opposite direction (e.g., Wang et al., 2015; Huang et al., 2018), the Bering land bridge played the key role for the spreading of seed plants, including crucifers, from Eurasia to the North American subcontinent (e.g., Carlsen et al., 2010; Wen et al., 2010; Karl and Koch, 2013; Jiang et al., 2019). Among the clades belonging to crucifer lineage I, some are endemic to the New World (Halimolobeae and Physarieae), others have a bi-continental distribution in Asia and America (Camelineae, Cardamineae, Crucihimalayeae, Descurainieae, Erysimeae, and Smelowskieae), but the Boechereae are confined to North America with only three species occurring in the Russian Far East. The bi-continental distribution of several tribes in the lineage I, and tribes of other crucifer lineages (e.g., Arabideae: Karl and Koch, 2013), makes the Bering land bridge the most plausible colonization route for several (and perhaps all) crucifer clades to North America.

Since the Halimolobeae are consistently retrieved as the sister clade to Boechereae (e.g., Beilstein et al., 2010; Nikolov et al., 2019) and Halimolobeae genomes resemble the ACK genome with *n* = 8, it is likely that the ancestral genomes of Boechereae and Halimolobeae arose from this common ancestor. The present-day geographic ranges of the two tribes differ, with Boechereae concentrated in the United States and Canada (barely extending into Mexico) and Halimolobeae generally more southern in distribution. In fact, Halimolobeae has two distinct centers of diversity, one extending from central Mexico to the southwestern United States, and the other in the Andes, from Ecuador to central Argentina (Bailey et al., 2007). The geographic ranges of the two tribes overlap in a narrow band stretching east to west along the United States/Mexico border. As both tribes have the closest phylogenetic affinity to the primarily Eurasian clades of lineage I, we propose that the MRCA of Boechereae/Halimolobeae reached North America via the Bering land bridge. Couvreur et al. (2010) estimated that the Boechereae and Halimolobeae diverged c. 8 mya (late Miocene) and their MRCA diverged from its predominantly Eurasian sister lineage during mid to early Miocene. The Bering land bridge connected northeastern Asia and northwestern North America from the Cretaceous until the Pliocene (Gladenkov et al., 2002; Jiang et al., 2019). In the latter study, rates of dispersal from Eurasia to North America were significantly elevated throughout the Oligocene and early Miocene (c. 34 to 16 mya), particularly around 26 to 24 mya. These time estimates broadly coincide with the origin and diversification of Brassicaceae lineage I in early Miocene (∼23 to 18 mya; Hohmann et al., 2015), which likely used the Bering land bridge to disperse from northeastern Asia to North America.

### Apomixis-Related Chromosomes in the Boechereae

Two heterochromatic chromosomes (*Het* and *Del*) have been previously described in *Boechera* apomicts (Kantama et al., 2007; Mandáková et al., 2015a). In eudiploid apomicts (2*n* = 14), a *Het* chromosome was identified as one of the Boe1 homologs (GBs A, C, and D). In aneuploid apomicts (2*n* = 15, 22), a centric fission partitioned *Het* chromosome to a larger *Het‘* (GBs A and C) and to a smaller *Del* (block D) chromosome (Mandáková et al., 2015b). In *P. cheiranthoides*, apomictic triploids and tetraploids contained a heterochromatic *Het* (GBs A, C, and D), but it was absent in diploid apomicts (Mandáková et al., 2020). This observation indicates that aposporic reproduction in *Phoenicaulis* is not associated with the presence of a *Het* chromosome. This is further corroborated herein by the apparent absence of a *Het* chromosome in two other aposporous apomicts, tetraploid *P. fremontii* and triploid *S. whitedii*.

### Autotetraploids in the Boechereae

While diploid or nearly diploid (2*n* = 15) species and hybrids seem to prevail in Boechereae (cf. BrassiBase^4^, accessed on 1 February 2020), apomictic triploid (2*n* = 21) or nearly triploid (2*n* = 22) hybrids are very common in *Boechera* (Schranz et al., 2005; Li et al., 2017) and recently discovered in *Phoenicaulis* (Mandáková et al., 2020) and *S. whitedii* (this study). Tetraploids (2*n* = 28) are rarely reported and in *Boechera* all cases of tetraploidy studied to date involve interspecific hybridization (i.e., allopolyploidy; see Windham et al., 2014; Li et al., 2017). Autotetraploidy was previously documented only in *P. cheiranthoides* (Mandáková et al., 2020), where the tetraploid cytotype is more common and widespread than either the diploid or triploid. Here we report two new cases of autotetraploidy in Boechereae, involving *C. douglasii* and *P. fremontii*. Hence, *bona fide* autotetraploids occur in three out of nine Boechereae genera. The autotetraploid species/cytotypes reproduce either sexually (*Cusickiella*) or, more frequently, by apomixis (*Phoenicaulis* and *Polyctenium*). Autopolyploids often experience irregular chromosome segregation due to the multivalent formation, but apomixis can potentially bypass such problematic meioses (Darlington, 1939; Stebbins, 1971; Comai, 2005; Cosendai et al., 2011). Indeed, while the analyzed tetraploid plants of *P. fremontii* show exclusive quadrivalent pairing (Figure 5), they appear to be fully fertile due to apospory (Figure 8). Hence, apomixis appears to be stabilizing reproduction in triploids and autotetraploids, which otherwise would suffer from semi-sterility due to chromosome pairing irregularities.

### Apomixis Originated Several Times Independently During Brassicaceae and Boechereae Diversification

Following the pioneering work on apomixis in *B. holboellii* [as *Arabis holboellii* Hornem. in Böcher (1951)], Mulligan (1966) reported its occurrence in *Erysimum* L. (Erysimeae) and Mosquin and Hayley (1966) documented possible asexual seed production in *Parrya* R. Brown (Chorisporeae). Mulligan and Findlay (1970) identified several species of *Draba* L. (Arabideae) that they inferred to be apomictic, and subsequent embryological and single seed flow cytometry analyses of one of these species (*Draba oligosperma* Hook.) were suggestive of apospory (Jordon-Thaden and Koch, 2012). Detailed cytological studies of *Draba*, *Erysimum*, and *Parrya* are needed to verify the regular occurrence of asexual seed production and the specific pathway involved. However, the available anecdotal evidence for this reproductive pathway occurring in four distantly related crucifer tribes (and its induction by hybridization in a fifth tribe, Brassiceae; Ellerstrom, 1983) suggests that apomixis has evolved independently multiple times within the family.

The Boechereae appear to be a “hotspot” for the origin and diversification of apomictic taxa. Our earlier publications have embryologically confirmed apomixis (either diplospory or apospory) in more than 20 diploid and triploid taxa of *Boechera* (Windham et al., 2015; Carman et al., 2019), and apospory in diploid, triploid, and tetraploid *Phoenicaulis* (Mandáková et al., 2020), and in diploid *Borodinia laevigata* (Muhl. ex Willd.) P. J. Alexander and Windham [as *Boechera laevigata* (Muhl. ex Willd.) Al-Shehbaz in Carman et al., 2019]. Here, two new genera are added to the list of aposporous apomicts, *Polyctenium* (tetraploid) and *Sandbergia* (triploid). Two genera of Boechereae (*Anelsonia* and *Yosemitea*) remain unsampled, and the exclusive occurrence of sexual reproduction in *Nevada* and *Cusickiella* must be confirmed by more extensive sampling. In summary, apomixis is now known to occur in five of the nine genera of Boechereae, and in two of these (*Phoenicaulis* and *Polyctenium*) it is the only reproductive pathway documented to date. Based on current sampling, *Sandbergia* exhibits equal proportions of apomictic (*S. whitedii*) and sexual reproduction (*S. perplexa*). Among *Boechera* species, apomictic taxa of hybrid origin greatly outnumber their sexual diploid progenitors (Li et al., 2017), while in *Borodinia*, sexual populations appear to predominate.

High frequency apospory (70% of ovules) was observed in man-made *Raphanus* L. × *Brassica* L. hybrids (Brassiceae) (Ellerstrom and Zagorcheva, 1977; Ellerstrom, 1983), which is consistent with wide hybridization occasionally inducing apomixis in otherwise sexual species (Carman, 1997). The long-term reproductive stability conferred by apomixis to sterile or semisterile inter-specific hybrids could provide novel genotypes with sufficient time (possibly hundreds of years) to fortuitously produce, by facultative sexual reproduction, recombinants that are more or less genomically stable (autopolyploidized, sensu Sybenga, 1996) and sexually fertile (Carman, 1997, 2007; Carman et al., 2019). If chromosome aberrations have occurred, the newly formed recombinant genomes might warrant specific or generic status. Hence, apomixis in genomically unstable taxa may facilitate (Carman, 1997; Horandl and Hojsgaard, 2012; Hojsgaard et al., 2014; Carman et al., 2019) rather than terminate (Darlington, 1939; Stebbins, 1971) speciation. That apomixis is prevalent in many large genera, e.g., among the rose, aster, and grass families (Carman, 1997; Hojsgaard et al., 2014), as well as in *Boechera*, supports this hypothesis.

## Data Availability Statement

All datasets generated for this study are included in the article/Supplementary Material.

## Author Contributions

TM, JC, and ML conceived the experiments. TM-O, PH, KA, and BP conducted the study and processed the data. ML, TM, JC, and MW wrote the manuscript. All authors have read and approved the final manuscript.

## Conflict of Interest

The authors declare that the research was conducted in the absence of any commercial or financial relationships that could be construed as a potential conflict of interest.

## Footnotes

1 https://brassibase.cos.uni-heidelberg.de/

2 http://www.arabidopsis.org

3 https://phytozome.jgi.doe.gov/pz/portal.html

4 https://brassibase.cos.uni-heidelberg.de/
