# Supplementary material for: Chromosomal Evolution and Apomixis in the Cruciferous Tribe Boechereae
Source: Front Plant Sci. 2020 May 28;11:514. doi: 10.3389/fpls.2020.00514 (PMC7270200; doi:10.3389/fpls.2020.00514)
Supplement: Supplementary file 1 [file Image_1.PDF]

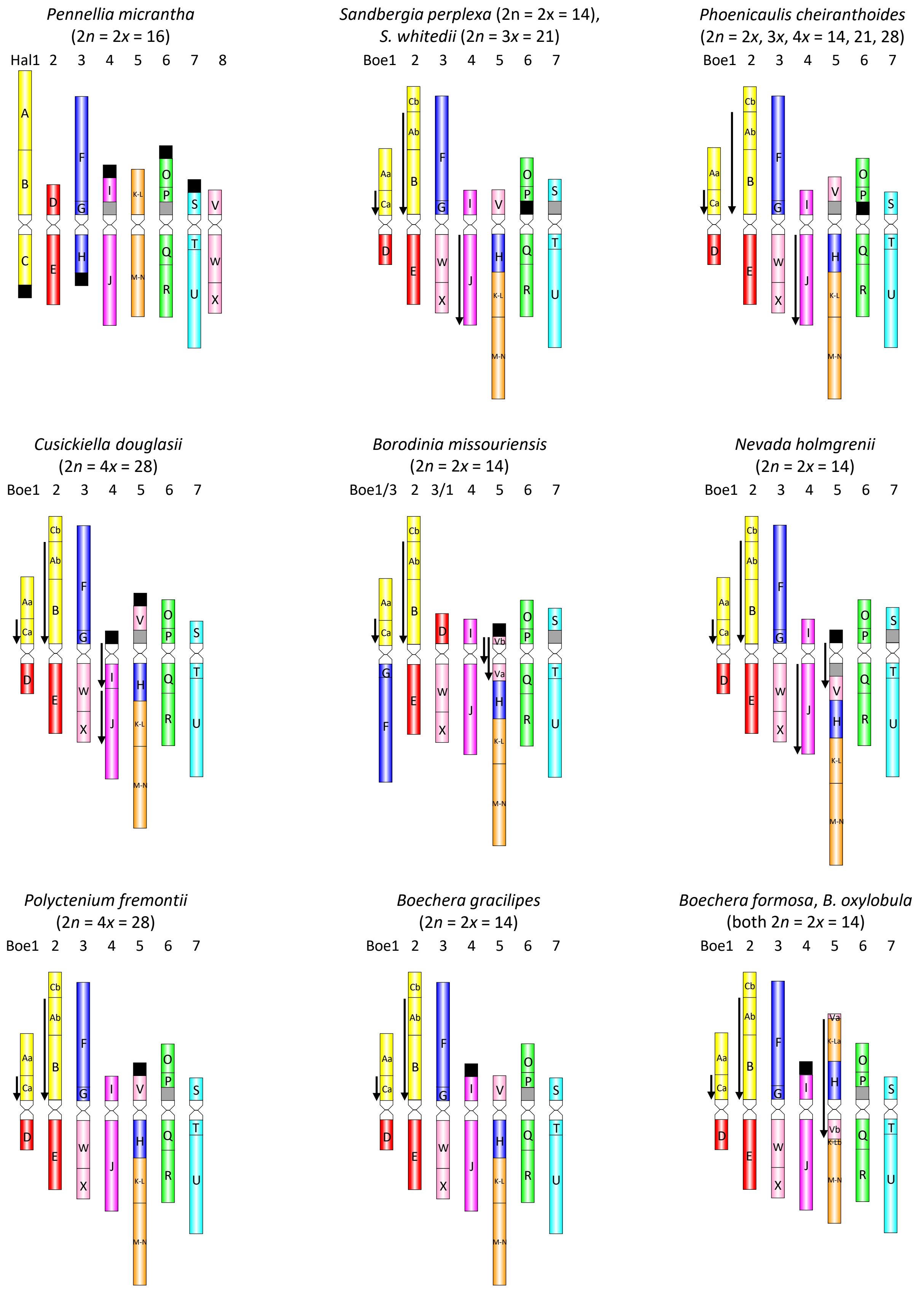

Supplementary Figure S1. Chromosomal position of 5S and 35S rDNA loci in karyotypes of Boechereae and Halimolobeae species analyzed. Color coding of 22 genomic blocks (GBs A to X) reflects their position on the eight ancestral chromosomes (AK1 to AK8) in the Ancestral Crucifer Karyotype. Blocks split into two segments are labeled as “a” and “b”. Downward-pointing arrows denote the inverse orientation of GBs compared to their position in the ACK represented here by the *Pennellia micrantha* genome. Grey squares: 5S rDNA loci, black squares: 35S rDNA loci.
